# Evolocumab attenuate pericoronary adipose tissue density via reduction of lipoprotein(a) in type 2 diabetes mellitus: a serial follow-up CCTA study

**DOI:** 10.1186/s12933-023-01857-w

**Published:** 2023-05-22

**Authors:** Meng-Meng Yu, Xin Zhao, Yin-Yin Chen, Xin-Wei Tao, Jun-Bo Ge, Hang Jin, Meng-Su Zeng

**Affiliations:** 1 Department of Radiology, Zhongshan Hospital, Fudan University, Shanghai Institute of Medical Imaging, No. 180 Fenglin Road, Xuhui District, Shanghai, 200032 China; 2 Department of Cardiology, Zhongshan Hospital, Fudan University, Shanghai Institute of Cardiovascular Diseases, National Clinical Research Center for Interventional Medicine, No. 180 Fenglin Road, Xuhui District, Shanghai, 200032 China; 3 Bayer Healthcare, No. 399, West Haiyang Road, Shanghai, 200126 China

**Keywords:** Coronary artery disease, Multidetector computed tomography, Pericoronary adipose tissue, Evolocumab, Lipoprotein(a)

## Abstract

**Background:**

Pericoronary adipose tissue (PCAT) density is a biomarker of vessel inflammation, which is supposed to be increased in patients with type 2 diabetes mellitus (T2DM). However, whether the coronary inflammation revealed by this novel index could be alleviated after evolocumab treatment in T2DM remains unknown.

**Methods:**

From January 2020 to December 2022, consecutive T2DM patients with low-density lipoprotein cholesterol ≥ 70 mg/dL on maximally tolerated statin and taking evolocumab were prospectively included. In addition, patients with T2DM who were taking statin alone were recruited as control group. The eligible patients underwent baseline and follow-up coronary CT angiography with an interval of 48-week. To render patients with evolocumab as comparable to those controls, a propensity-score matching design was used to select the matched pairs with a 1:1 ratio. Obstructive lesion was defined as the extent of coronary artery stenosis ≥ 50%; the numbers inside the brackets were interquartile ranges.

**Results:**

A total of 170 T2DM patients with stable chest pain were included [(mean age 64 ± 10.6 [range 40–85] years; 131 men). Among those patients, 85 were in evolocumab group and 85 were in control group. During follow-up, low-density lipoprotein cholesterol (LDL-C) level (2.02 [1.26, 2.78] vs. 3.34 [2.53, 4.14], *p* < 0.001), and lipoprotein(a) (12.1 [5.6, 21.8] vs. 18.9 [13.2, 27.2], *p* = 0.002) were reduced after evolocumab treatment. The prevalence of obstructive lesions and high-risk plaque features were significantly decreased (*p* < 0.05 for all). Furthermore, the calcified plaque volume were significantly increased (188.3 [115.7, 361.0] vs. 129.3 [59.5, 238.3], *p* = 0.015), while the noncalcified plaque volume and necrotic volume were diminished (107.5 [40.6, 180.6] vs. 125.0 [65.3, 269.7], *p* = 0.038; 0 [0, 4.7] vs. 0 [0, 13.4], *p* < 0.001, respectively). In addition, PCAT density of right coronary artery was significantly attenuated in evolocumab group (− 85.0 [− 89.0, − 82.0] vs. − 79.0 [− 83.5, − 74.0], *p* < 0.001). The change in the calcified plaque volume inversely correlated with achieved LDL-C level (r =  − 0.31, *p* < 0.001) and lipoprotein(a) level (r =  − 0.33, *p* < 0.001). Both the changes of noncalcified plaque volume and necrotic volume were positively correlated with achieved LDL-C level and Lp(a) (*p* < 0.001 for all). However, the change of PCAT_RCA_ density only positively correlated with achieved lipoprotein(a) level (r = 0.51, *p* < 0.001). Causal mediation analysis revealed Lp(a) level mediated 69.8% (*p* < 0.001) for the relationship between evolocumab and changes of PCAT_RCA_.

**Conclusions:**

In patients with T2DM, evolocumab is an effective therapy to decrease noncalcified plaque volume necrotic volume, and increase calcified plaque volume. Furthermore, evolocumab could attenuate PCAT density, at least in part, via the reduction of lipoprotein(a).

**Supplementary Information:**

The online version contains supplementary material available at 10.1186/s12933-023-01857-w.

## Introduction

The prevalence of diabetes mellitus is increasing worldwide due to a growing obesity epidemic and an aging population [1]. Approximately 90–95% of the diabetes population are classified as type 2 diabetes mellitus (T2DM). Patients with T2DM have a higher risk of cardiovascular events and death than those without diabetes [2, 3]. According to previous studies, T2DM is associated with premature atherosclerosis and increased risk of coronary artery disease (CAD), which is the most common cause of mortality in patients with diabetes [4]. The T2DM is featured by increased concentration of circulatory cytokines as a result of inflammation [5], which plays a vital role in the development and progression of coronary atherosclerosis. Therefore, suppression of coronary inflammation among patients with T2DM is of great importance.

Currently, proprotein convertase subtilisin/kexin type 9 (PCSK9) inhibitors are a new class of drugs [6], which may benefit patients with high risk of atherosclerotic cardiovascular disease, such as diabetes mellitus. It not only produce a strong low-density lipoprotein cholesterol (LDL-C) reduction, but also contribute to a modest reduction of lipoprotein(a) [Lp(a)] [7]. Lp(a) is a preferential lipoprotein carrier of oxidized phospholipids (OxPLs) that lead to vascular inflammation [8, 9], which associated with coronary atherosclerosis and unfavorable clinical outcome. Currently, whether PCSK9 inhibitor helps to alleviate coronary inflammation in patients with T2DM remains unknown.

Recently, increased CT attenuation in pericoronary adipose tissue (PCAT) density assessed by coronary computed tomography angiography (CCTA) has been proposed as a reliable quantitative marker of vessel inflammation [10]. According to previous studies, increased PCAT density is associated with the presence of high-risk plaque features [11, 12], which correlated with the progression of CAD and incidence of major adverse cardiovascular events (MACEs) [13]. Furthermore, an observational CCTA study implied that PCAT density was higher in diabetic patients than that in non-diabetic patients regardless of coronary stenotic severity and high-risk plaque characteristics [14]. However, among patients with T2DM, whether the coronary inflammation revealed by this novel index could be alleviated after PCSK9 inhibitor treatment still unclear.

A recent case study [15] have reported that the PCSK9 inhibitor could contribute to modulating arterial wall inflammatory with reduction of Lp(a) level. Therefore, we hypothesized that PCSK9 inhibitor helps to attenuate the vessel inflammation in patients with T2DM, which would be noninvasively assessed by PCAT density at CCTA examination. The aim of our study was to investigate the changes of PCAT density as determined by CCTA after evolocumab treatment in patients with T2DM.

## Materials and methods

### Patient sample

From January 2020 to December 2022, consecutive patients who were diagnosed as T2DM [16] with stable chest pain and met the inclusion criterion below were prospectively included: (a) patients with a fasting low-density lipoprotein cholesterol (LDL-C) ≥ 70 mg/dL (1.8 mmol/L) on a background of maximally tolerated statin therapy; (b) patients were taking evolocumab (140 mg every 2-week) at baseline visit according to the 2019 ESC guidelines [17]. The exclusion criteria were: (a) patients had been treated with evolocumab previously; (b) contraindicated with the usage of iodine contrast median; (c) primary cardiomyopathy (hypertrophic, dilated, and restrictive); (d) severe hepatic and renal insufficiency; (e) patients underwent coronary revascularization during 48 weeks follow-up; (f) refused to participate; (g) inadequate image quality of CCTA.

In addition, to further explore the effect of evolocumab on the plaque modification, patients with T2DM who were taking statin alone were recruited as control group within the same period. The exclusion criterion were the same as the evolocumab group.

The eligible patients underwent baseline and follow-up CCTA examination with an interval of 48-week. Institutional review board approval was obtained for the present study, and informed consent was obtained from all patients. The LDL-C, total cholesterol (TC), high-density lipoprotein cholesterol (HDL-C), triglycerides (TG), Lp(a), C-reactive protein (CRP) and hemoglobin A1c (HbA1c) at baseline and over 48-week were compared.

### CCTA protocol

The third generation dual source CT (SOMATOM Force, Siemens Healthineers, Germany) was used for data acquisition. Nitroglycerin was given sublingually in all patients before CCTA scan. Calcium score was firstly performed to calculate the calcification burden of each epicardial vessels. CCTA was performed by using a bolus tracking technique, with regions of interest placed in the descending aorta. A bolus of contrast media (40–50 mL, 370 mg iodine/mL, Ultravist, Bayer) was injected into antecubital vein at the rate of 4–5 mL/s, followed by a 40 mL saline flush by using dual-barrel power injector. Prospective ECG-triggered sequential acquisition was used in all patients with the triggering window covering from end-systolic to mid-diastolic phase (from 35 to 75% of R–R interval), with collimation = 96 × 0.6 mm, reconstructed slice thickness = 0.75 mm, reconstructed slice interval = 0.5 mm, rotation time = 250 ms and application of automated tube voltage and current modulation (CAREKv, CAREDose 4D, Siemens Healthineers, Germany). The tube voltage was 70, 80, 90, 100, 110 or 120 kVp. The reference tube current was set as 320 mAs and the reference tube voltage was set as 100 kVp. All CCTA data were reconstructed with a smooth kernel (Bv 40) and third generation iterative reconstruction technique (ADMIRE, strength level 3, Siemens Healthineers, Germany). The same acquisition parameters were used for baseline and follow-up CCTA.

### Image reconstruction and analysis of CCTA

Data were transferred to an offline workstation (Syngo via, Siemens Healthineers) for further analyses. Coronary arteries with a diameter ≥ 2 mm were evaluated on every coronary artery and its branches. Any segment with the presence of atherosclerosis, defined as any tissue ≥ 1 mm^2^ within or adjacent to the lumen that could be discriminated from surrounding pericardial, epicardial fat, or lumen, and identified in > 2 planes, were all included for analysis. For longitudinal comparisons of CCTA, baseline and follow-up coronary lesions were matched using fiduciary landmarks (e.g., side branches, distance from the ostium) and analyzed side-by-side. Obstructive CAD was defined as the extent of coronary artery stenosis ≥ 50%. Quantified plaque characterization was performed semi-automatically by using a dedicated plaque analysis software (Coronary Plaque Analysis, version 5.0.2, Siemens Healthineers). Parameters were measured as follow: (1) Agatston score; (2) high-risk plaque features, including low-attenuation plaque, positive remodeling, spotty calcification and napkin-ring sign; (3) total plaque volume, noncalcified plaque volume, calcified plaque volume and necrotic volume (CT < 30HU); (4) PCAT density of right coronary artery (RCA), left anterior descending artery (LAD), and left main trunk (LMT). All of the quantitative parameters were per-patient based analysis. Details were provided in Additional file 1.

### CT quantification of PCAT

A dedicated PCAT attenuation analysis software (Coronary FAI Analysis, version 5.0.2, Siemens Healthineers, Germany) was used for quantification. PCAT attenuation was the mean CT attenuation of adipose tissue, which was within a radial distance from the outer vessel wall equal to the diameter of the target vessel.

To calculate patients-based PCAT attenuation, we measured three segments (RCA, LAD and LMT). PCAT attenuation analysis of RCA involved the proximal 10–50 mm of the vessel, excluding the most proximal 10 mm to avoid the effects of the aortic wall [10]. In the LAD, we analyzed the proximal 40 mm of LAD. In the LMT, 5 mm proximal to the bifurcation were analyzed. In addition, our study adjusted the tube voltages using a conversion factor as previously reported [18]. Accordingly, PCAT_RCA,_ PCAT_LAD_ and PCAT_LMT_ were recorded.

Two cardiovascular radiologists (with 37 years and 9 years of experience in cardiac imaging, respectively) who were blinded to the information of medication in use, independently analyzed CCTA data. The mean values of parameters were used for further analysis.

### Statistical analysis

Statistical analysis was performed with R version 3.3.0 software (Vienna, Austria) and MedCalc Statistical Software (MedCalc Software bvba; version 15.2.2). One-sample Kolmogorov–Smirnov test was used to check the assumption of normal distribution. Continuous variables were expressed as mean ± SD, while median and quartiles were used otherwise. Student’s t test was used for normally distributed data, whereas Mann–Whitney U test was used for the data that were not normally distributed. Categorical variables were reported as count (%) and compared using chi-square test. The interobserver agreements of all parameters were examined for intra-class correlation coefficients (ICCs). The correlations analysis were assessed by Pearson test when data were normally distributed or Spearman's test when data were not normally distributed. To balance the baseline characteristics of the evolocumab group versus control group, we utilized 1:1 nearest-neighbor propensity score matching (PSM) with a multivariable logistic regression model. Details were provided in Additional file 1. Statistical significance was defined as a two-sided *p* < 0.05.

The causal-steps approach [19] was used for assessing whether the effect of the evolocumab on PCAT density was carried through the reduction of serum Lp(a) level. Then, the bootstrapping mediation method [20] was applied to quantify the mediation effects mediated by Lp(a) level on the association between evolocumab and PCAT. Covariates (age, gender, body mass index, hypertension, LDL-C, total plaque volume, noncalcified plaque volume, calcified plaque volume, necrotic volume, as well as high-risk plaque features) were included in models both in the causal-steps approach and bootstrapping mediation method.

## Results

### Patient characteristics

**Fig. 1:**
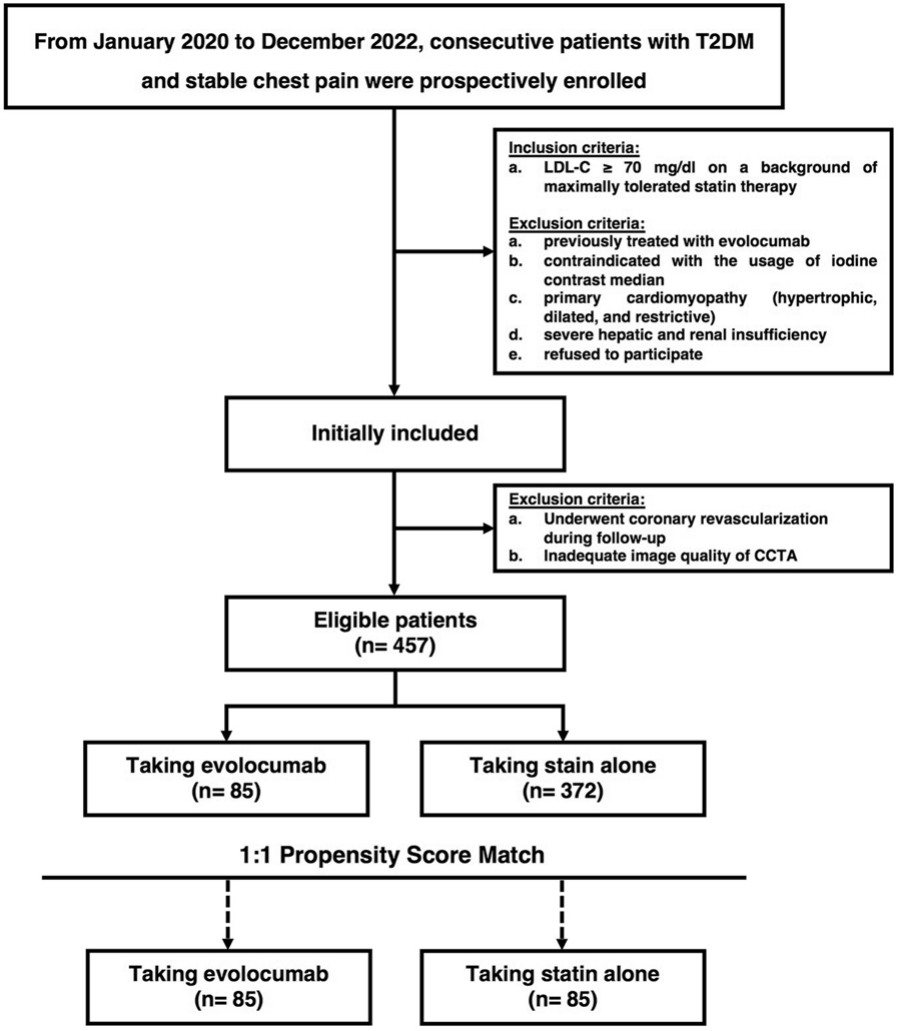
Flow chart of inclusion and exclusion. CCTA, coronary computed tomography angiography; LDL-C, low-density lipoprotein cholesterol; T2DM, type 2 diabetes mellitus

Initially, 85 patients were in evolocumab group and 372 patients were in control group (details in Additional file 1: Table S1). PSM was performed to balance potential confounders between two groups. Finally, a total of 170 patients (mean age 64 ± 10.6 [range 40–85] years; 131 men) were included (details in Fig. 1). Among those patients, 85 were in evolocumab group and 85 were in control group, respectively. There were no significant differences in clinical characteristics between the two groups at baseline (*p* > 0.05 for all, details in Table 1). The details of the pretest and posttest medication use are provided in Tables 1 and 2.

**Table 1:** Clinical characteristics

Characteristic	Evolocumab	Control	*p* value
	n = 85	n = 85	
Age (y)^b^	64 ± 8.3	65 ± 11.2	0.545
Men, n (%)	67 (78.8)	64 (75.2)	0.715
BMI (kg/m^2^)^b^	24.2 ± 3.3	23.5 ± 3.8	0.158
Risk factors, n (%)			
Hypertension	62 (72.9)	59 (69.4)	0.734
Hyperlipidemia	29 (34.1)	32 (37.6)	0.749
Current or past tobacco use	15 (17.6)	19 (22.3)	0.565
Clinical presentation, n (%)			0.819
Typical angina	22 (25.9)	24 (28.2)	
Atypical angina	28 (32.9)	30 (35.3)	
Non-anginal pain	35 (41.1)	31 (36.4)	
Baseline statin use^a^, n (%)			0.886
High intensity	10 (11.8)	9 (10.6)	
Moderate intensity	56 (65.9)	59 (69.4)	
Low intensity	19 (22.3)	17 (20.0)	
Baseline insulin in use, n (%)	19 (22.3)	17 (20)	0.851
History of myocardial infarction	18 (21.1)	21 (24.7)	0.715
History of revascularization	20 (23.5)	22 (25.8)	0.858
Heart rate during CT scan	68 ± 6.9	67 ± 6.7	0.508
β blocker use during CT scan	68 (80.0)	71 (83.5)	0.691
Tube voltage of CT acquisition, n (%)			0.915
70 kVp	5 (5.8)	7 (8.2)	
80 kVp	21 (24.7)	18 (21.1)	
90 kVp	25 (29.4)	23 (27.0)	
100 kVp	19 (22.3)	23 (27.0)	
110 kVp	9 (10.8)	10 (11.7)	
120 kVp	6 (7.0)	4 (4.7)	

Unless otherwise specified, data are numbers of patients, with percentages in parentheses

BMI, body mass index

^a^Baseline statin use was defined as defined as subject treated with statin therapy at the time of screening. High-intensity statins: atorvastatin ≥ 40 mg, rosuvastatin ≥ 20 mg, simvastatin ≥ 80 mg daily. Moderate-intensity statins: atorvastatin 10 to < 40 mg, rosuvastatin 5 to < 20 mg, simvastatin 20 to < 80 mg daily. Low-intensity statins: atorvastatin < 10 mg, rosuvastatin < 5 mg, simvastatin < 20 mg daily

^b^Numbers are means ± standard deviations

**Table 2:** Laboratory findings and medication in use in evolocumab group and control group

	Evolocumab (n = 85)			Control (n = 85)			*p* value between groups	
	Baseline	Follow-up	*p*	Baseline	Follow-up	*p*	Baseline	Follow-up
Laboratory findings^a^								
TC (mmol/L)	4.56 (2.78, 6.89)	3.32 (2.18, 4.37)	< 0.001	4.48 (2.98, 6.69)	3.81 (2.62, 5.89)	0.052	0.914	0.016
HDL-C (mmol/L)	1.38 (1.23, 1.65)	1.41 (1.55, 1.80)	0.869	1.32 (1.11, 1.52)	1.35 (1.21, 1.62)	0.301	0.622	0.099
LDL-C (mmol/L)	3.34 (2.53, 4.14)	2.02 (1.26, 2.78)	< 0.001	3.23 (2.54, 4.02)	3.18 (2.63, 3.79)	0.904	0.666	< 0.001
TG (mmol/L)	1.56 (1.23, 2.11)	1.38 (1.13, 1.58)	0.005	1.47 (1.28, 1.98)	1.33 (1.13, 1.73)	0.088	0.922	0.499
Lp(a) (mg/dL)	18.9 (13.2, 27.2)	12.1 (5.6, 21.8)	0.002	17.6 (12.6, 27.5)	18.9 (9.5, 28.2)	0.918	0.650	0.001
HbA1c (%)	6.6 (5.9, 7.3)	6.8 (6.1, 7.4)	0.473	6.7 (6.0, 7.4)	6.9 (6.1, 7.4)	0.472	0.376	0.443
CRP (mg/L)	3.1 (1.9, 3.9)	2.9 (2.5, 3.2)	0.531	2.8 (1.9, 3.4)	2.9 (2.4, 3.5)	0.543	0.284	0.867
Medication in use, n (%)								
ACEI/ARB	49 (57.6)	51 (60.0)	0.876	47 (55.2)	50 (58.8)	0.756	0.877	1.000
β-Blocker	33 (38.8)	35 (41.1)	0.875	35 (41.1)	39 (45.9)	0.642	0.875	0.643
Nitrates	29 (34.1)	31 (36.5)	0.873	32 (37.6)	36 (42.3)	0.638	0.749	0.530

Unless otherwise specified, data are numbers of patients, with percentages in parentheses

ACEI, Angiotensin-converting enzyme inhibitor; ARB, Angiotensin receptor blocker; CRP, C-reactive protein; HbA1c, Hemoglobin A1c; HDL-C, High density lipoprotein cholesterol; LDL-C, Low density lipoprotein cholesterol; Lp(a), Lipoprotein(a); TC, Total cholesterol; TG, Triglyceride

^a^Numbers are medians, with interquartile ranges in parentheses

### Baseline and follow-up laboratory findings

Table 2 shows the laboratory findings between groups. There were no significant differences between groups at baseline. After 48-week treatment, TC (3.32 [2.18, 4.37] vs. 4.56 [2.78, 6.89], *p* < 0.001), LDL-C (2.02 [1.26, 2.78] vs. 3.34 [2.53, 4.14], *p* < 0.001), TG (1.38 [1.13, 1.58] vs. 1.56 [1.23, 2.11], *p* = 0.005) and Lp(a) (12.1 [5.6, 21.8] vs. 18.9 [13.2, 27.2], *p* = 0.002) were significantly decreased in evolocumab group. However, in this group, there were no significant difference between the baseline and follow-up with regards to HDL-C, HbA1c and CRP (*p* > 0.05 for all). In the control group, no significant difference were observed with respect to TC, HDL-C, LDL-C, TG, Lp(a), HbA1c and CRP (*p* > 0.05 for all).

After 48-weeks, the evolocumab group showed lower TC (3.32 [2.18, 4.37] vs. 3.81 [2.62, 5.89], *p* = 0.016), LDL-C (2.02 [1.26, 2.78] vs. 3.18 [2.63, 3.79], *p* < 0.001) and Lp(a) (12.1 [5.6, 21.8] vs. 18.9 [9.5, 28.2], *p* = 0.001) than the control group.

### Baseline and follow-up CCTA parameters

**Fig. 2:**
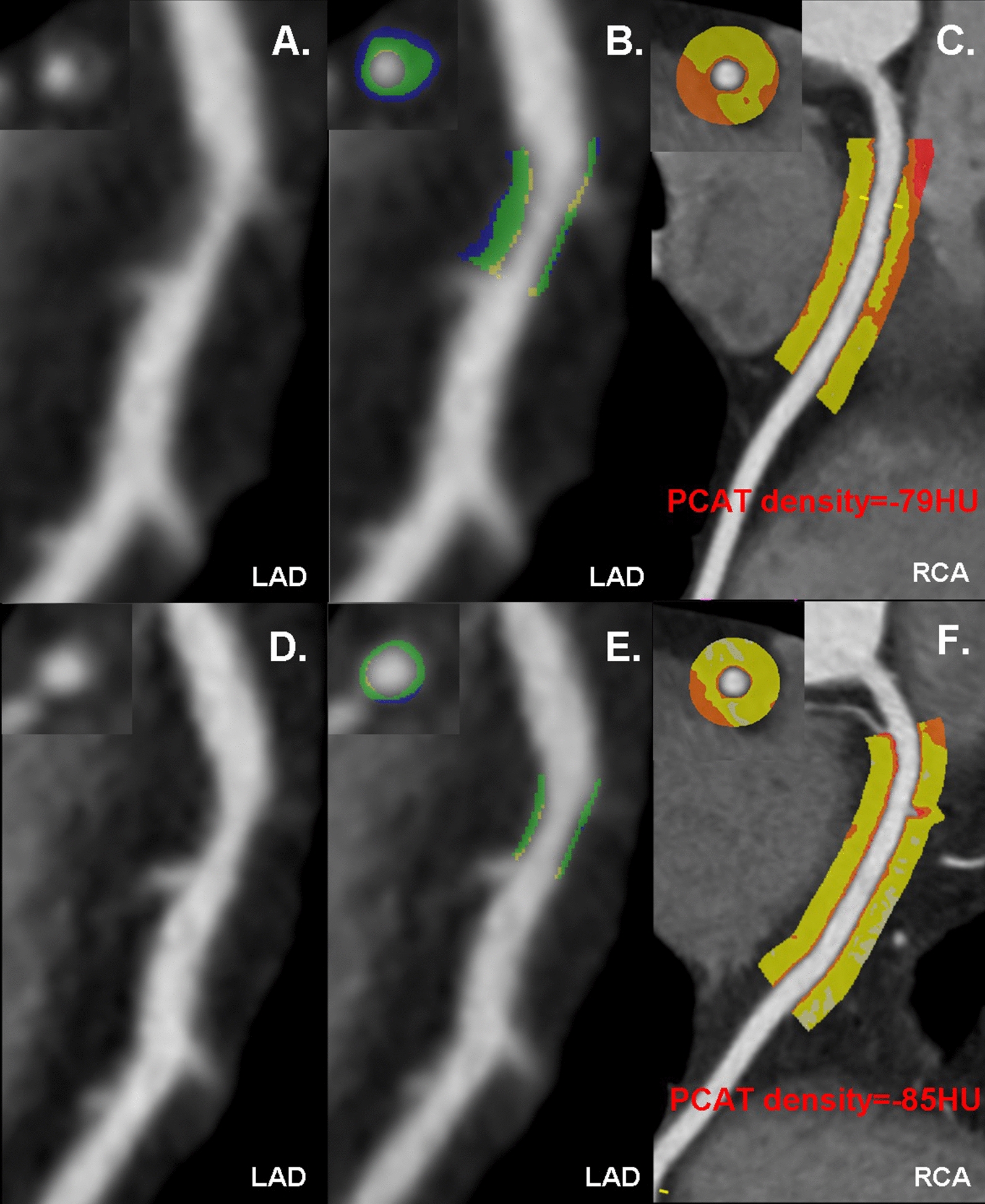
A representative case of a type 2 diabetes patients treated with evolocumab for 48-week. **A**–**C** The baseline LDL-C and Lp(a) level was 3.6 mmol/L and 64 mg/dL. Baseline CCTA revealed noncalcified plaque with moderate stenosis of middle left anterior descending artery. The total plaque volume was 132.2 mm^3^ and the PCAT density of right coronary artery was − 79HU. **D**–**F** After 48-week treatment, both the LDL-C and Lp(a) level were reduced to 1.5 mmol/L and 19 mg/dL, respectively. The follow-up CCTA revealed the lesion regression with mild stenosis. The follow-up total plaque volume decreased to 11.8 mm^3^ and PCAT density of right coronary artery attenuated to − 85HU. CCTA, coronary computed tomography angiography; HU, hounsfield unit; Lp(a), lipoprotein(a); LDL-C, low-density lipoprotein cholesterol; PCAT, pericoronary adipose tissue

Changes in lesion characteristics between the baseline and follow-up findings are displayed in Table 3. At baseline, no significant difference were observed between groups. In the evolocumab group, compared with those at baseline, follow-up CCTA revealed increased total Agatston score but did not achieve the significant level (251.5 [157.6, 384.0] vs. 178.2 [96.5, 344.6], *p* = 0.057). The percentage of obstructive CAD decreased (35.2% vs. 51.8%, *p* = 0.044). In terms of high-risk plaque features, the prevalence of low-attenuation plaque and positive remodeling were significantly reduced (both *p* < 0.05, details in Table 3). In addition, the calcified plaque volume were significantly increased (188.3 [115.7, 361.0] vs. 129.3 [59.5, 238.3], *p* = 0.015), while the noncalcified plaque volume (107.5 [40.6, 180.6] vs. 125.0 [65.3, 269.7], *p* = 0.038) and necrotic volume (0 [0, 4.7] vs. 0 [0, 13.4], *p* < 0.001) were decreased. The total plaque volume showed mildly increased but did not get the significant level (289.2 [195.8, 582.3] vs. 223.4 [154.2, 573.7], *p* = 0.194). Furthermore, PCAT density was significantly attenuated in evolocumab group (PCAT_RCA_: − 85.0 [− 89.0, − 82.0] vs. − 79.0 [− 83.5, − 74.0], *p* < 0.001; PCAT_LAD_: − 83.1 [− 87.2, − 80.1] vs. − 76.9 [− 81.1, − 71.9], *p* < 0.001; PCAT_LMT_: − 82.3 [− 86.3, − 79.2] vs. − 76.3 [− 80.6, − 71.3], *p* < 0.001; Fig. 2 and Additional file 1: Fig. S1).

**Table 3:** Baseline and follow-up CCTA findings in evolocumab group and control group: patient based analysis

	Evolocumab (n = 85)			Control (n = 85)			*p* value between groups	
	Baseline	Follow-up	*p*	Baseline	Follow-up	*p*	Baseline	Follow-up
Total Agatston score^a^	178.2 (96.5, 344.6)	251.5 (157.6, 384.0)	0.057	214 (83.0, 421)	228.1 (118.5, 424.2)	0.536	0.900	0.333
Obstructive CAD, n (%)	44 (51.8)	30 (35.2)	0.044	42 (49.4)	39 (45.9)	0.758	0.878	0.211
High-risk plaque features, n (%)								
Low attenuation plaque	33 (38.8)	19 (22.3)	0.031	37 (43.5)	34 (40.0)	0.755	0.640	0.020
Positive remodeling	45 (52.9)	31 (36.4)	0.044	47 (55.2)	42 (49.4)	0.622	0.877	0.121
Spotty calcification	15 (17.6)	13 (15.3)	0.836	16 (18.8)	14 (16.4)	0.840	1.000	1.000
Napkin-ring sign	26 (30.1)	16 (18.8)	0.109	29 (34.1)	22 (25.9)	0.315	0.743	0.357
Atheroma volume (mm^3^)^a^								
Total plaque volume	223.4 (154.2, 573.7)	289.2 (195.8, 582.3)	0.194	218.4 (119. 3, 457.5)	238.6 (172.7, 457.5)	0.420	0.576	0.191
Calcified plaque volume	129.3 (59.5, 238.3)	188.3 (115.7, 361.0)	0.015	103.5 (37.8, 233.5)	126.0 (62.0, 214.5)	0.097	0.281	0.030
Noncalcified plaque volume	125.0 (65.3, 269.7)	107.5 (40.6, 180.6)	0.038	147.2 (70.3, 273.2)	117.2 (55.0, 194.5)	0.475	0.715	0.043
Necrotic volume	0 (0, 13.4)	0 (0, 4.7)	< 0.001	0 (0, 26.2)	0 (0, 21.1)	0.713	0.611	0.047
PCAT_RCA_ (HU)^a^	− 79.0 (− 83.5, − 74.0)	− 85.0 (− 89.0, − 82.0)	< 0.001	− 79.0 (− 86.5, − 72.5)	− 81.0 (− 90.0, − 76.5)	0.102	0.772	0.015
PCAT_LMT_ (HU)^a^	− 76.3 (− 80.6, − 71.3)	− 82.3 (− 86.3, − 79.2)	< 0.001	− 75.4 (− 82.5, − 70.1)	− 78.7 (− 86.0, − 71.0)	0.172	0.980	0.001
PCAT_LAD_ (HU)^a^	76.9 (− 81.1, − 71.9)	− 83.1 (− 87.2, − 80.1)	< 0.001	76.3 (− 84.2, − 70.6)	79.1 (− 87.4, − 72.5)	0.076	0.773	0.013

Unless otherwise specified, data are numbers of patients, with percentages in parentheses

CAD, Coronary artery disease; HU, Hounsfield unit; LAD, left anterior descending artery; LMT, left main trunk; PCAT, Pericoronary adipose tissue; RCA, right coronary artery

^a^Numbers are medians, with interquartile ranges in parentheses

Among the control group, there were no significant difference between baseline and follow-up in terms of total Agatston score, total plaque volume, calcified plaque volume, noncalcified plaque volume, necrotic volume, high-risk plaque features as well as PCAT density (*p* > 0.05 for all).

The interobserver agreement for total Agatston score, total plaque volume, calcified plaque volume, noncalcified plaque volume, necrotic volume, high-risk plaque features, and PCAT density were excellent (Additional file 1: Table S2).

### Correlation analysis

**Fig. 3:**
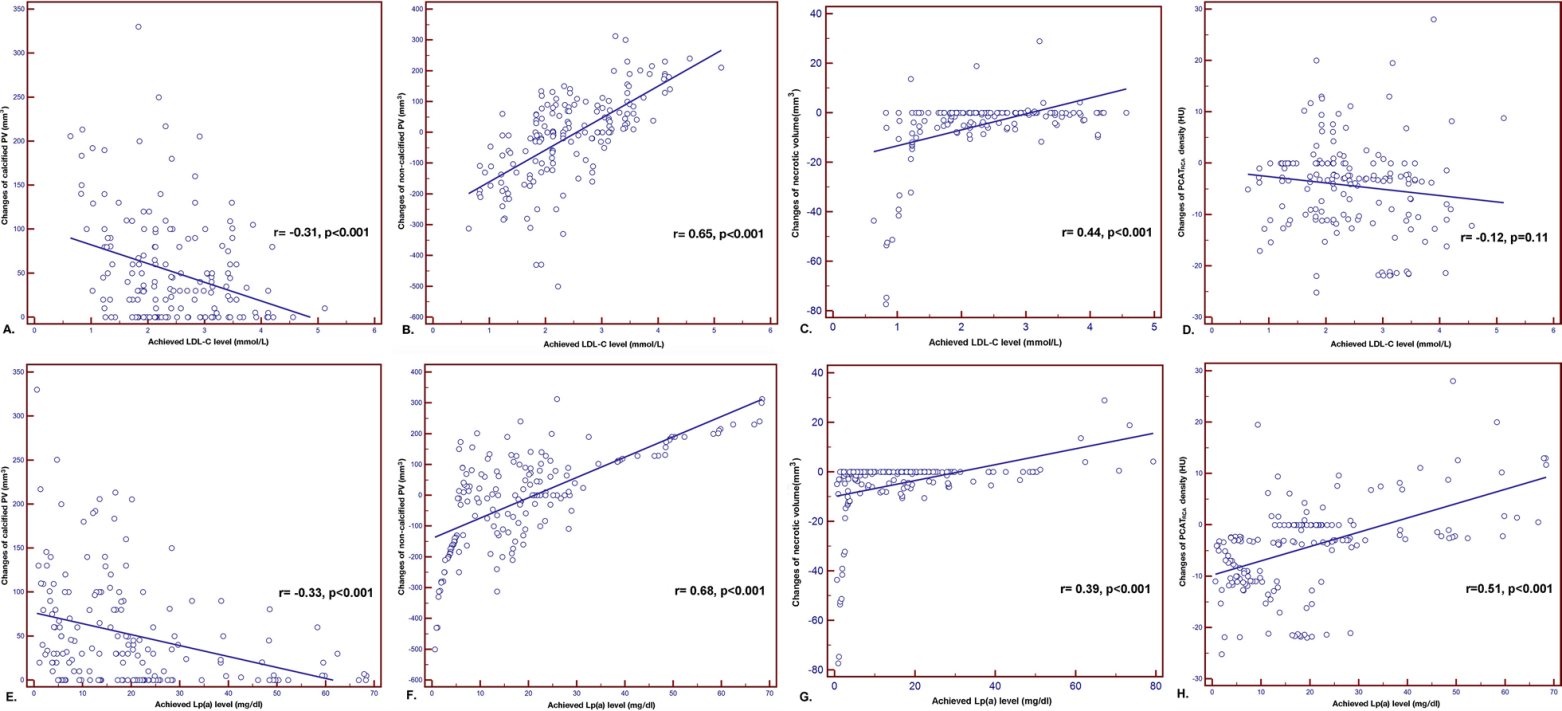
The correlation analysis. **A**–**D** The change in the calcified plaque volume inversely correlated with achieved LDL-C level (r =  − 0.31, *p* < 0.001); There was a positive correlation between the change in noncalcified plaque volume and achieved LDL-C level (r = 0.65, *p* < 0.001); Similar finding were observed in the relationship between necrotic volume and achieved LDL-C level (r = 0.44, *p* < 0.001). However, the change of PCAT_RCA_ density did not correlated with achieved LDL-C level (r =  − 0.12, *p* = 0.11). **E**–**H** There was a negative association between the change of calcified plaque volume and achieved Lp(a) level (r =  − 0.33, *p* < 0.001); The change in the noncalcified plaque volume positively associated with achieved Lp(a) level (r = 0.68, *p* < 0.001); Similar finding were observed in the relationship between necrotic volume and achieved LDL-C level (r = 0.39, *p* < 0.001). In addition, the change in PCAT_RCA_ density positively correlated with achieved Lp(a) level (r = 0.51, *p* < 0.001). CCTA, coronary computed tomography angiography; Lp(a), lipoprotein(a); LDL-C, Low-density lipoprotein cholesterol; PCAT, pericoronary adipose tissue; RCA, right coronary artery

The change in the calcified plaque volume inversely correlated with achieved LDL-C level (r =  − 0.31, *p* < 0.001) and Lp(a) level (r =  − 0.33, *p* < 0.001). There was a positive correlation between the change in noncalcified plaque volume and achieved LDL-C level (r = 0.65, *p* < 0.001) and Lp(a) (r = 0.68, *p* < 0.001). Furthermore, the changes of necrotic volume positively associated with achieved LDL-C level (r = 0.44, *p* < 0.001) and Lp(a) (r = 0.39, *p* < 0.001) either. However, the change of PCAT_RCA_ density only positively correlated with achieved Lp(a) level (r = 0.51, *p* < 0.001) but not LDL-C level (r =  − 0.12, *p* = 0.11; Fig. 3). Similar finding were observed in PCAT_LAD_ and PCAT_LMT_ (Details in Additional file 1: Fig. S2).

### Causal mediation analysis

**Fig. 4:**
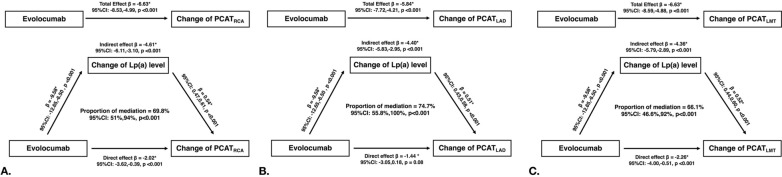
Mediation analysis of the change of Lp(a) level for the relationship between evolocumab treatment and change of PCAT density. **A** The total effect of evolocumab on change of PCAT_RCA_ density is − 6.63 (95% CI − 8.53, − 4.99, *p* < 0.001). The changes of Lp(a) level had a significant indirect effect (β = − 4.61, 95% CI − 6.11, − 3.10, *p* < 0.001) and mediated 69.8% (*p* < 0.001) for the relationship evolocumab treatment and change of PCAT_RCA_ density. **B** The changes of Lp(a) level had a significant indirect effect (β = − 4.40, 95% CI − 5.83, − 2.95, *p* < 0.001) and mediated 74.7% (*p* < 0.001) for the relationship evolocumab treatment and change of PCAT_LAD_ density. **C** The changes of Lp(a) level had a significant indirect effect (β = - 4.36, 95% CI − 5.79, − 2.89, *p* < 0.001) and mediated 66.1% (*p* < 0.001) for the relationship between evolocumab treatment and change of PCAT_LMT_ density. *Adjusted for age, gender, body mass index, hypertension, low-density lipoprotein cholesterol, total plaque volume, noncalcified plaque volume, calcified plaque volume, necrotic volume, as well as high-risk plaque features. CI, confidence interval; LAD, left anterior descending; LMT, left main trunk; Lp(a), lipoprotein(a); LDL-C, low-density lipoprotein cholesterol; PCAT, pericoronary adipose tissue; RCA, right coronary artery

Causal-steps approach showed: (i) evolocumab treatment was significantly associated with the reduction of Lp(a) level (β = − 9.58, 95% CI − 12.65 to − 6.50, *p* < 0.001); (ii) the change of Lp(a) level was correlated with the improvement of PCAT_RCA_ (β = 0.54, 95% CI 0.47–0.61, *p* < 0.001). The bootstrapping method showed: (i) the total effect of evolocumab on PCAT_RCA_ is − 6.63 (95% CI − 8.53 to − 4.99, *p* < 0.001); (ii) In addition, the change of Lp(a) level had a significant indirect effect (β =  − 4.61, 95% CI − 6.11 to − 3.10, *p* < 0.001) and mediated 69.8% (*p* < 0.001) for the relationship between evolocumab and changes of PCAT_RCA_. The mediation effects were also found in PCAT_LAD_ and PCAT_LMT_ (Details in Fig. 4)_._

### Safety and tolerability

All of the patients adhere well to evolocumab. During the treatment of evolocumab, 5 (5.8%) patients presented with mild injection-site reactions, including injection-site erythema and muscle pain. None of the patients discontinued evolocumab due to injection-site reactions.

## Discussion

In this prospective observational study, 48-week evolocumab treatment resulted in a significant regression of noncalcified plaque volume and necrotic volume, and an elevation of calcified plaque volume. A change in the characteristics of high-risk plaque was also present, with a reduction in the prevalence of low-attenuation plaque and positive remodeling. In addition, the PCAT density could be attenuated after evolocumab treatment, which was partially through the reduction of Lp(a) level. Furthermore, our study demonstrated excellent LDL-C-lowering tolerance, and safety of evolocumab.

Several studies have shown the effect of PCSK-9 inhibitor on coronary atherosclerosis. Based on a serial optical coherence tomography study of non–ST-segment elevation myocardial infarction patients [21], combination of evolocumab and statin leads to a greater increase in minimum fibrous cap thickness and decrease in maximum lipid arc than stain monotherapy. Serial intravascular ultrasound [22, 23] and optical coherence tomography [21] imaging studies have demonstrated that incremental lowering of LDL-C to very low levels with the PCSK-9 inhibitor produces regression of percent atheroma volume in statin-treated patients. Our results was consistent with previous findings. Although, the current study revealed an elevation of Agatston score and increasement of calcified plaque volume, which could portend worse prognosis [24, 25]. We suggest that interpretation of calcification progression should be stratified by lipid treatment. Inducing calcification of plaques may be one of the mechanisms by which evolocumab exert a positive effect in reducing the risk of MACEs. This hypothesis remains to be proven in future study.

In addition to the burden of coronary atherosclerosis and characteristics of high-risk plaque, PCAT density also have prognostic significance. Previous studies have reported that higher PCAT density was associated with an increased risk of cardiac mortality and poor prognosis [13]. Another study also found that CT attenuation increased around culprit lesions compared with nonculprit lesions in patients with acute coronary syndrome [11], emphasizing the clinical value of PCAT analysis assessed by CCTA. Therefore, attenuating PCAT density in patients with high risk of cardiovascular events, such as patients with T2DM, could improve the prognosis of clinical outcome.

Our study is the first to found that that the reduction of LDL-C level did not change PCAT density despite a decrease in the plaque volume. The change of PCAT density, at least in part, was attributed to the decrease of Lp(a) level after 48-week evolocumab treatment. Furthermore, the mediation analysis showed the indirect effect mediated by Lp(a) level was about 70%. Structurally, Lp(a) is an LDL-like particle to which apo(a) is covalently bound, the latter carrying proinflammatory oxidized phospholipids. Thus, Lp(a) could stimulate coronary inflammation in addition to plaque growth due to cholesterol accumulation. A prior study have demonstrated that inactivating Lp(a) therapies benefit to suppress inflammatory reaction in patients with elevated Lp(a) level [26], which further supported our results. Our study suggested that PCSK-9 inhibitor therapy contribute to alleviating coronary inflammation assessed by PCAT.

The clinical implication of the current study lies in using CCTA derived PCAT density for serial follow-up of medically treated coronary lesions in patients with T2DM. Vascular inflammation might precede the changes of luminal stenosis and geometrical features of coronary plaques [27]. Therefore, before the improvement of the extent of coronary stenosis and high-risk plaque features visualized by CCTA, the regression of active vascular inflammation may have already happened and could be captured by PCAT. Thus, PCAT density is potentially a more sensitive and useful parameter over conventional plaque features to monitor the treatment effect by evolocumab. Regarding its clinical significance, CCTA will become the arbiter of treatment success by means of routine quantification of pericoronary inflammation.

Our study had limitations. First, it was a single-center study with relatively small samples. However, our study is a preliminary study and data on this topic were limited. Second, all of the patients was not followed up after the second CCTA, therefore it was not possible to determine the correlation of PCAT density change and MACEs. Third, only 11.2% patients were taking high-intensity statin in the current study, because the statin intolerance is more common in Asia than those in other countries [28]. Future multiethnic studies are needed to confirm our results. Forth, whether increasing coronary calcification would affect the clinical outcome in patients with lipid treatment remains unknown. Fifth, the LDL-C level at follow-up in the evolocumab group was relatively high compared to previous studies with PSCK-9 inhibitors. However, all of the patients included in our study were those with T2DM, which were different with the majority of previous studies. In addition, the patient sample in the current study was a relative small sample size. Future multicenter studies with larger sample size and longer follow-up period are warranted to investigate the relationship between PCAT improvement and clinical outcome.

In conclusion, evolocumab treatment produces favorable changes to plaque composition, including noncalcified plaque volume regression and calcified plaque volume progression, in patients with type 2 diabetes mellitus. Furthermore, it contribute to attenuate PCAT density, assessed by CCTA examination, which maybe ascribe to the reduction of Lp(a).

## Supplementary Information

**Additional file 1.** Evolocumab attenuate pericoronary adipose tissue density via reduction of lipoprotein(a) in type 2 diabetes mellitus: a serial follow-up CCTA study.

## Data Availability

Data of the current study will be available upon request to corresponding author, if complying with the patient privacy policy of local hospital.

## Abbreviations

CAD: Coronary artery disease
CCTA: Coronary computed tomography angiography
CRP: C-reactive protein
HbA1c: Hemoglobin A1c
HDL-C: High-density lipoprotein cholesterol
LDL-C: Low-density lipoprotein cholesterol
Lp(a): Lipoprotein(a)
PCAT: Pericoronary adipose tissue
PCSK9: Proprotein convertase subtilisin/kexin type 9
TC: Total cholesterol
T2DM: Type 2 diabetes mellitus
TG: Triglycerides
