# Supplementary material for: Evolocumab attenuate pericoronary adipose tissue density via reduction of lipoprotein(a) in type 2 diabetes mellitus: a serial follow-up CCTA study
Source: Cardiovasc Diabetol. 2023 May 22;22:121. doi: 10.1186/s12933-023-01857-w (PMC10204214; doi:10.1186/s12933-023-01857-w)
Supplement: Supplementary file 1 — Additional file 1. Evolocumab attenuate pericoronary adipose tissue density via reduction of lipoprotein(a) in type 2 diabetes mellitus: a serial follow-up CCTA study. [file 12933_2023_1857_MOESM1_ESM.docx]

**Supplemental material**

**CCTA image analysis**

High-risk plaque features, including low-attenuation plaque, positive remodeling, spotty calcification and napkin-ring sign were measured. Detailed definitions of the above parameters were given as the following: 1) Low-attenuation plaque was defined as any voxel <30 HU within a coronary plaque, using a dedicated plaque analysis software (Coronary Plaque Analysis, version 5.0.2, Siemens Healthineers); 2) Remodeling index was defined as a maximal lesion vessel diameter divided by proximal reference vessel diameter, with PR defined as a remodeling index ≥ 1.1; 3) Spotty calcification was defined by an intra-lesion calcific plaque < 3 mm in length that comprised < 90 degrees of the lesion circumference; 4) Napkin-ring sign was characterized by a plaque core with low attenuation areas on CT surrounded by a rim-like area of higher attenuation.

Subtype plaque volumes were calculated based on the Hounsfield units (HU) values: necrotic volume (< 30 HU), noncalcified plaque (30–130HU), and calcified plaque (> 130 HU).

**Statistical analysis**

To balance the baseline characteristics of the evolocumab group versus control group, we utilized 1:1 nearest-neighbor propensity score matching (PSM) with a multivariable logistic regression model. The following characteristics: age, sex, body mass index, hypertension, hyperlipidemia, current or past tobacco use, clinical presentation, baseline medication in use (including statin, insulin, ACEI/ARB, β-Blocker, and nitrates), LDL-C, TC, HDL-C, TG, Lp(a), CRP, HbA1c, total plaque volume, PCAT density and high-risk plaque features were included into the PSM analysis. The Caliper, which represents the maximum standardized difference between matched subjects, was set to 0.02.

The sample size was estimated using PASS (Power Analysis and Sample Size) (PASS version 11.0, UT, USA). Because there was no published literature on the change of PCATRCA at 48-week follow-up after evolocumab treatment. We determined the sample size based on our preliminary results of our own data. Before the enrollment of the current study, we have retrospectively reviewed 92 patients (not the current study) who underwent baseline and follow-up CCTA. Among those patients, 31 patients were taking evolocumab and 61 patients only taking stain treatment. The interval between baseline and follow-up CCTA were 48 weeks. According to our preliminary results, the change of PCATRCA were -6.4 ± 7.4 HU and -2.3 ± 6.1 HU in patients taking evolocumab and in patients only taking stain treatment respectively. Thus, a necessary and sufficient sample size of 58 patients in each group can achieve 90% power to detect a difference between the groups. Considering a 10% drop-out rate, we needed sample sizes of 64 in each group, respectively. Then, a total of 170 patients were prospectively enrolled and followed up for 48-weeks. This sample size achieved >90% power to detect a difference between the groups.

**Online Table 1. Clinical characteristics**

| Characteristic | Evolocumab  n= 85 | Control  n= 372 | *P* Value |
| --- | --- | --- | --- |
| Age (y)* | 64.2 ± 8.3 | 56.6 ± 12.4 | <0.001 |
| Men, n(%) | 67(78.8) | 232(62.3) | 0.006 |
| BMI (kg/m^2^) * | 24.2 ± 3.3 | 23.3 ± 3.7 | 0.041 |
| Risk factors, n(%) |  |  |  |
| Hypertension | 62(72.9) | 189(50.8) | <0.001 |
| Hyperlipidemia | 29(34.1) | 92(24.7) | 0.102 |
| Current or past tobacco use | 15(17.6) | 71(19.0) | 0.878 |
| Clinical presentation, n(%) |  |  | 0.432 |
| Typical angina | 22(25.9) | 79(21.2) |  |
| Atypical angina | 28(32.9) | 112(30.1) |  |
| Non-anginal pain | 35(41.1) | 181(48.6) |  |
| Baseline statin use^#^, n(%) |  |  | 0.961 |
| High intensity | 10(11.8) | 45(12.0) |  |
| Moderate intensity | 56(65.9) | 249(66.9) |  |
| Low intensity | 19(22.3) | 78(20.9) |  |
| Baseline insulin in use, n(%) | 19(22.3) | 58(15.6) | 0.179 |
| History of myocardial infarction | 18(21.1) | 51(13.7) | 0.117 |
| History of revascularization | 20(23.5) | 68(18.2) | 0.339 |
| Baseline laboratory findings^$^ |  |  |  |
| TC (mmol/L) | 4.56[2.78, 6.89] | 3.81[2.73, 5.82] | 0.016 |
| HDL-C (mmol/L) | 1.38[1.23, 1.65] | 1.34[1.21, 1.64] | 0.636 |
| LDL-C (mmol/L) | 3.34[2.53, 4.14] | 2.92[2.30, 3.66] | 0.005 |
| TG (mmol/L) | 1.56[1.23, 2.11] | 1.22[0.83, 1.85] | 0.001 |
| Lp(a) (mg/dl) | 18.9[13.2, 27.2] | 17.7[10.8, 27.2] | 0.408 |
| HbA1c (%) | 6.6[5.90, 7.30] | 6.30[5.80, 7.00] | 0.055 |
| CRP (mg/L) | 3.1[1.90, 3.90] | 2.90[2.40. 3.45] | 0.540 |
| Medication in use, n (%) |  |  |  |
| ACEI/ARB | 49(57.6) | 165(44.3) | 0.036 |
| β-Blocker | 33(38.8) | 140(37.6) | 0.936 |
| Nitrates | 29(34.1) | 134(36.0) | 0.838 |

Note.—Unless otherwise specified, data are numbers of patients, with percentages in parentheses.

Abbreviations: ACEI= Angiotensin-converting enzyme inhibitor; ARB =Angiotensin receptor blocker; BMI= body mass index; CRP= C-reactive protein; HbA1c = Hemoglobin A1c; HDL-C= High density lipoprotein cholesterol; LDL-C= Low density lipoprotein cholesterol; Lp (a)= Lipoprotein(a); TC= Total cholesterol; TG= Triglyceride.

**^*^** Numbers are means ± standard deviations.

**^$^**Numbers are medians, with interquartile ranges in parentheses.

**^#^** Baseline statin use was defined as defined as subject treated with statin therapy at the time of screening. High-intensity statins: atorvastatin ≥40 mg, rosuvastatin ≥20 mg, simvastatin ≥80 mg daily. Moderate-intensity statins: atorvastatin 10 to <40 mg, rosuvastatin 5 to <20 mg, simvastatin 20 to <80 mg daily. Low-intensity statins: atorvastatin <10 mg, rosuvastatin <5 mg, simvastatin <20 mg daily.

*Numbers are means ± standard deviations.

**Online Table 2. Inter-observer Reproducibility**

| **Variables** | **ICC** | **95%CI** | **p value** |
| --- | --- | --- | --- |
| Total Agatston score | 0.98 | 0.97 to 1.00 | <0.001 |
| Low attenuation plaque | 0.94 | 0.90 to 0.94 | <0.001 |
| Positive remodeling | 0.92 | 0.90 to 0.94 | <0.001 |
| Spotty calcification | 0.94 | 0.90 to 0.96 | <0.001 |
| Napkin-ring sign | 0.94 | 0.92 to 0.95 | <0.001 |
| Total plaque volume | 0.92 | 0.90 to 0.94 | <0.001 |
| Calcified plaque volume | 0.97 | 0.95 to 0.98 | <0.001 |
| Noncalcified plaque volume | 0.93 | 0.90 to 0.94 | <0.001 |
| Necrotic volume | 0.95 | 0.90 to 0.98 | <0.001 |
| PCAT_RCA_ | 0.95 | 0.92 to 0.97 | <0.001 |
| PCAT_LMT_ | 0.96 | 0.93 to 0.98 | <0.001 |
| PCAT_LAD_ | 0.94 | 0.92 to 0.97 | <0.001 |

Abbreviations: CI= confidence interval; LAD= left anterior descending; LMT= left main trunk; PCAT= Pericoronary adipose tissue; RCA= right coronary artery; ICC= Intraclass correlation coefficient


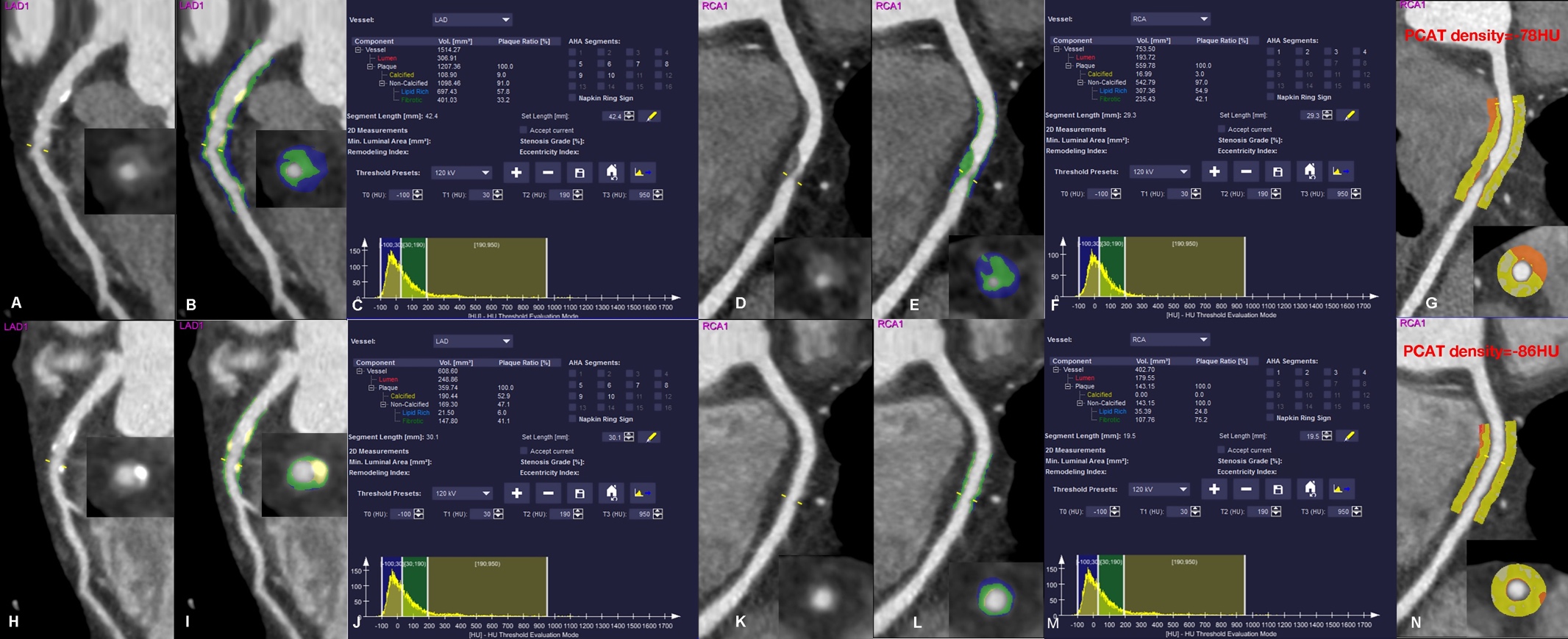


**Online Figure 1: A representative case of a type 2 diabetes patients treated with evolocumab for 48-week.**

(A)-(G): The baseline LDL-C and Lp(a) level was 3.4 mmol/L and 51 mg/dL, respectively. Baseline CCTA revealed mixed plaque with moderate stenosis from the end of the left main trunk to the middle segment of left anterior descending artery, and noncalcified plaque with severe stenosis within the middle segment of right coronary artery. The total plaque volume, noncalcified plaque volume, calcified plaque volume and necrotic volume was 1767.14 mm^3^, 1640.73 mm^3^, 125.89 mm^3^ and 1004.79 mm^3^. The PCAT density of right coronary artery attenuated was -78 Hu.

(H)-(N): After 48-week treatment, both the LDL-C and Lp(a) level were reduced to 1.7 mmol/L and 11 mg/dL, respectively. The follow-up CCTA revealed the lesion regression with minimal stenosis. The total plaque volume, noncalcified plaque volume, and necrotic volume decreased to 502.89 mm^3^, 312.45 mm^3^ and 56.89 mm^3^. The calcified plaque volume increased to 190.44 mm3. Furthermore, the PCAT density of RCA attenuated to -86 Hu.

Abbreviation: CCTA= coronary computed tomography angiography; HU= hounsfield unit; Lp(a)= lipoprotein(a); LDL-C= low-density lipoprotein cholesterol; PCAT= pericoronary adipose tissue


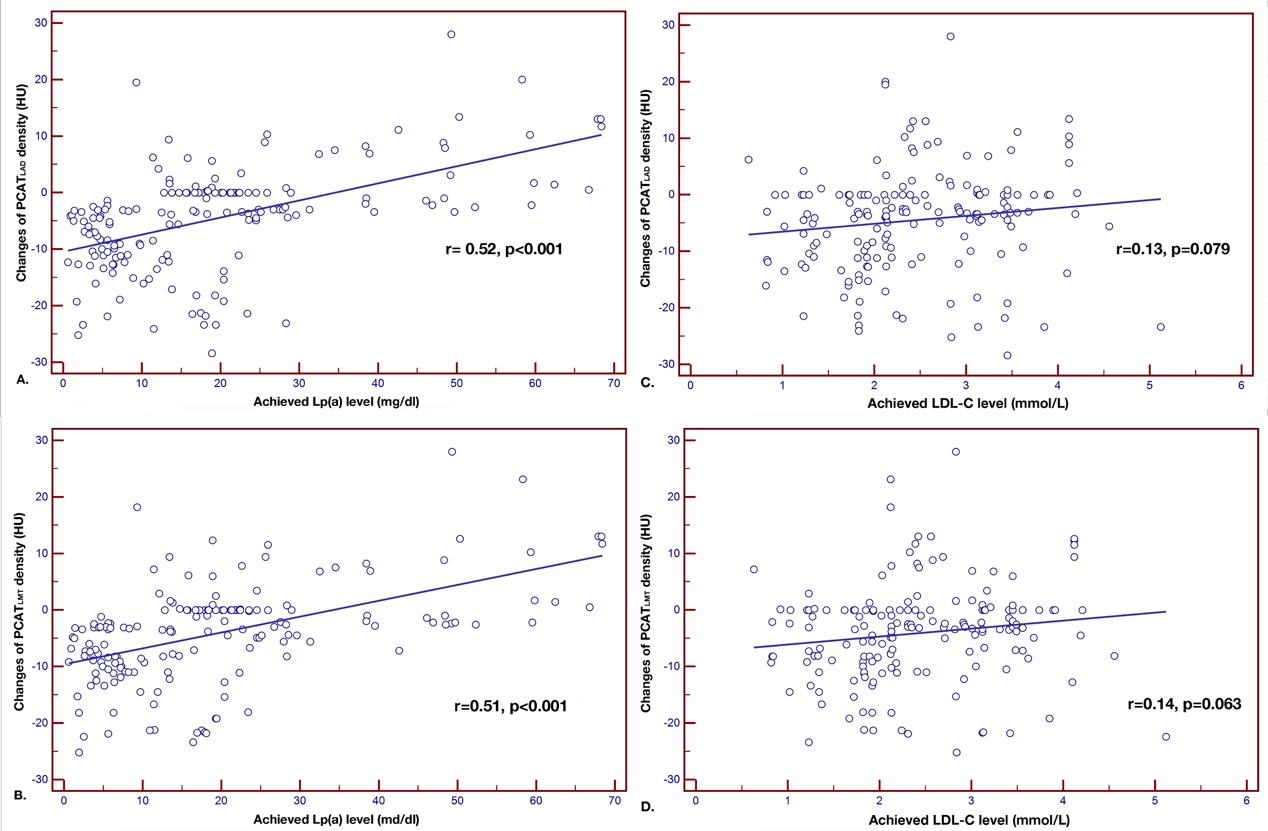


**Online figure 2: Correlation analysis between PCAT, achieved Lp(a) levels and LDL-C levels.**

Abbreviation: HU= Hounsfield unit; LAD= left anterior descending; LDL-C= low-density lipoprotein cholesterol; LMT= left main trunk; Lp(a)= lipoprotein(a); PCAT= pericoronary adipose tissue
